# The REALISE score: a new statistically validated scoring system to assess the severity of anal fissures

**DOI:** 10.1007/s10151-021-02459-y

**Published:** 2021-05-13

**Authors:** A. Picciariello, P. Lobascio, L. Spazzafumo, M. Rinaldi, R. Dibra, G. Trigiante, R. Laforgia, A. Pezzolla, D. F. Altomare

**Affiliations:** 1 Surgical Unit “M. Rubino” Department of Emergency and Organ Transplantation, University Aldo Moro of Bari, Bari, Italy; 2 Agenzia Regionale Sanitaria (The Regional Agency for Health), Marche Region, Ancona, Italy; 3 Unit of Laparoscopic Surgery, Department of Emergency and Organ Transplantation, University Aldo Moro of Bari, Bari, Italy

**Keywords:** Anal fissure, Scoring system, Reliability, Inter-/intraobserver agreement, Repeatability

## Abstract

**Background:**

Anal fissure (AF) is a common, painful disease that strongly affects patients’ quality of life, however, no scoring system to assess the severity of AF is available in the literature. The aim of this study was to set up and validate a reliable scoring system to quantify the severity of AF, to be used in prospective trials comparing the efficacy and the outcomes of surgical or medical treatments.

**Methods:**

The study was conducted on patients with acute or chronic AF and a control group in a tertiary centre for coloproctology in June 2020–September 2020. Two researchers independently carried out a structured interviewer-led questionnaire at two different time points (T1/T2). The questionnaire consisted of five items selected according to the most commonly reported symptoms for AF: the item pain, was scored from 0 to 10 using a visual analogue scale, and quality of life, duration of pain, use of painkillers, and bleeding were scored from 1 to 5 using Likert-scale questions. The scoRing systEm for AnaL fIsSurE (REALISE) score was the sum of the points. Patients with AF and a control group of patients with haemorrhoids, anal fistula, or obstructed defecation syndrome entered the study. Main outcome measures were reliability, inter-/intraobserver agreement, and repeatability.

**Results:**

One hundred and fifty well-matched patients (75 with AF and 75 controls) were enrolled. A significant difference was found between the mean REALISE score for patients with AF and controls (*p* < 0.001). The two REALISE scores were highly correlated (*r* = 0.99). The coefficient of repeatability was 1.45 in T1 and 1.18 in T2.

**Conclusions:**

The REALISE score may have an important role in the assessment and management of AF, in grading the severity of AF and comparing results of different treatments.

## Introduction

Anal fissure (AF) is a very common anorectal condition which equally affect both sexes [1] and is often associated with elevated internal anal sphincter pressure [2, 3]. The exact aetiology of this condition is still debated although several factors can play a role in fissure formation, such as anal trauma during the passage of hard stools, surgical trauma, local irritation in case of diarrhea and anoreceptive intercourse [4].

Although AF is a benign disease, it has a strong impact on patients’ quality of life especially when it is chronic, symptomatic and often unresponsive to any medical treatment for several months [5]. The main complaint leading these patients to consult the proctologist is the intense sharp pain that follows defecation and that can last several minutes to hours. In more than 70% of patients with AF bleeding (appearing as traces of bright red blood on the toilet paper or streaking the surface of the stool) may occur [6].

Approximately, 90% of AF are located in the midline posteriorly as this is the weaker point of the anoderm where a tear can occur and its low tendency to heal spontaneously seems to be related to the lower anodermal blood flow in this region worsened by anal sphincter spasm [7].

In most cases, the diagnosis of AF is suggested by the patients’ description of symptoms and can be confirmed by a simple physical examination of the anus [8]. In fact, anoscopy cannot be performed in most of the cases because of the severe pain experienced by the patient.

In the past two decades, several medical and surgical treatments have been proposed and have provided satisfactory outcomes regarding anal pain and healing rate [9–11].

However, no studies quantify the severity of the fissure in a reliable manner.

Several factors contribute to the severity of this condition including duration of pain, need to take painkillers, bleeding and effects on quality of life but a disease-specific, validated and easy to use severity index has not yet been proposed.

The aim of this paper was to propose and validate a reliable scoring system to quantify the severity of AF to be used in prospective trials comparing the efficacy and the outcomes of surgical or medical treatments.

## Materials and methods

### Patients

Patients with acute or chronic AF and controls, matched for sex and age, were enrolled in a tertiary centre for coloproctology in June 2020–September 2020 after the approval of the local Ethics Committee. Control group patients had II–III degree haemorrhoids or anal fistula or obstructed defecation syndrome. Exclusion criteria were pregnancy, patients with cancer or human immunodeficiency virus, Irritable bowel syndrome, previous anal surgery, psychiatric disorders, inability to sign or understand the informed consent. The control group included patients complaining similar anal symptoms due to different diseases instead of healthy subjects to evaluate the discriminatory power of the scoRing systEm for AnaL fIsSurE (REALISE) score. Written informed consent was obtained from all study participants.

### Questionnaire

Two researchers independently carried out a structured interviewer-led questionnaire at two different time points (T1 and T2), at an interval ranging between 1–2 h for the first time and 7–9 h for the second time. All the questionnaires were administered within 1 day because of the possible changes in the symptoms after the onset of the any treatment. The interview was carried out by telephone for the patients not able to return to the clinic. The questionnaire consisted of five items selected according to the most commonly reported symptoms for AF: the item pain, was scored from 0 to 10 using a visual analogue scale (VAS), the remaining four (quality of life duration of the pain, pain killers pill intake and bleeding) were scored from 1 to 5 points using a Likert-scale questions (Table 1). The REALISE score was the sum of all points, with a maximum possible of 30 points and a minimum of 4 points.

**Table 1:** The scoRing systEm for AnaL fIsSurE (REALISE) questionnaire

Variable	Score										
Q1: How severe is the anal pain (VAS)	0	1	2	3	4	5	6	7	8	9	10
	1			2		3		4		5	
Q2: How long does the pain last after the defecation?	Less than 1 h			> 1 to ≤ 2 h		> 2 to ≤ 3 h		> 3 to ≤ 4 h		> 4 h	
Q3: Do you take NSAIDs or other painkillers?	Never			Rarely (once a week)		Sometimes (2–3 times a week)		Often (from four times a week to every day)		Always (> 1 time in a day)	
Q4: How often does bleeding from the anus occur?	Never			Rarely (≤ 25% of defecations)		Sometimes (> 25 ≤ 50% of defecations)		Often (> 50% ≤ 75% of defecations)		Always (> 75% of defecations)	
Q5: How much does this disease impact your QoL?	No impact			Slightly		Moderately		Considerably		Severely	
Total score: Q1 + Q2 + Q3 + Q4 + Q5											

*VAS* visual analogue scale, *QoL* quality of life, *NSAIDS* nonsteroidal anti-inflammatory drugs

### Statistical analysis

To measure the agreement between the two researchers’ results the Kappa coefficient were calculated for each single item and the REALISE score.

The Bland and Altman [12] plot was used to assess the repeatability of total score by comparing the REALISE score assigned by the two researchers and the coefficient of repeatability (CR) was calculated as 1.96 times the standard deviations of the differences between the two measurements (T1 and T2).

Because the scores for each item was ordinal, the nonparametric Mann–Whitney *U* test was used to compare the median REALISE score between controls and patients.

A *p* value of < 0.05 was considered statistically significant.

The sensitivity and specificity of the score was performed by the receiver operating characteristic (ROC) curve analysis.

The sample size of 75 patients per arm was calculated to provide approximately 83% of power to detect a medium effect size of 0.5 on variation of the REALISE score between the AF group and the control group, considering an α error probability of 0.043 and a *β* error probability of 0.172, applying the nonparametric Mann–Whitney *U* two-tailed test with a 5% significance level.

## Results

A total of 150 patients (mean age 47 years, SD ± 13.2) were enrolled in the study. Half of them (AF group) had AF. Out of 75 patients belonging to the control group, 51 (68%) had haemorrhoids, 17 (22.6%) had an anal fistula and 7 (9.4%) had obstructed defecation. AF was chronic in 47% of patients. AF and control groups were well matched for age and sex.

The mean age of the AF group and control group was, respectively, 48.3 (SD ± 12.4) and 45.9 (SD ± 14.1) years.

There were a total of 77 (51.3%) females, 39 (50.6%) in the AF group (Table 2).

**Table 2:** Contingency table for sex (*p* = 0.87, *χ*2 = 0.027)

Sex	Groups		Total	
	Control	Anal fissure		
F	*N* = 38	*N* = 39	*N* = 77	
% of total F	49.4	50.6	100	
% in each group	50.7	52.0	51.3	
M	*N* = 37	*N* = 36	*N* = 73	
% of total M	50.7	49.3	100	
% in each group	49.3	48.0	48.7	

The REALISE scores of the two researchers were distributed normally and were highly correlated (*r* = 0.99). The correlation between the scores assigned to each item ranged from 0.96 to 0.99 (Tables 3 and 4).

**Table 3:** Correlations between each item assigned by two researchers and Kappa coefficients at T1

	Item 1	Item 2	Item 3	Item 4	Item 5
Correlation coefficient (*r*)	0.98	0.99	0.99	0.98	0.96
Kappa coefficient	0.77	0.95	0.95	0.92	0.86

**Table 4:** Correlations between each item assigned by two researchers and Kappa coefficients at T2

	Item 1	Item 2	Item 3	Item 4	Item 5
Correlation coefficient (*r*)	0.99	0.99	0.99	0.98	0.97
Kappa coefficient	0.85	0.98	0.95	0.94	0.87

**Fig. 1:**
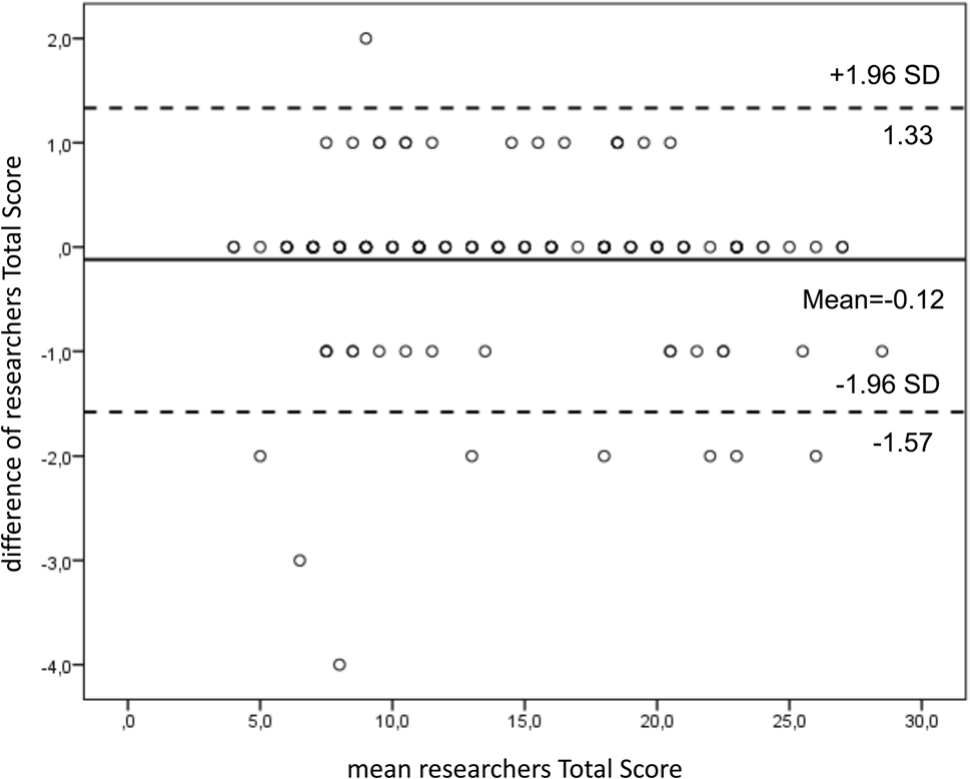
Bland and Altman plot difference between the two researchers’ scores vs. average of the two REALISE scores at T1

**Fig. 2:**
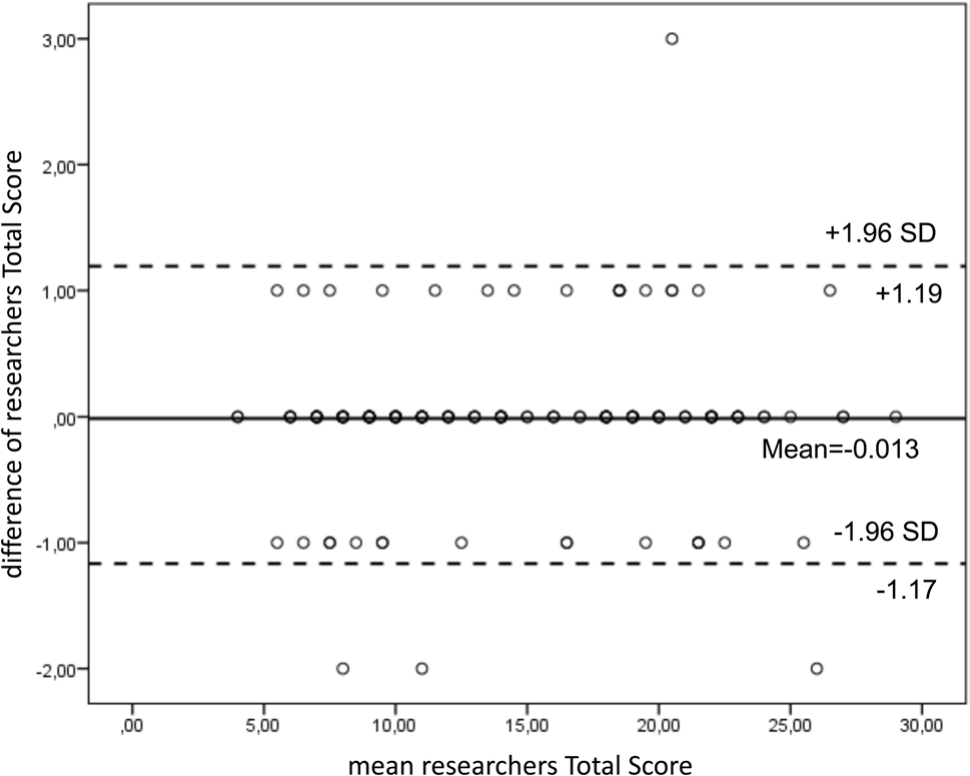
Bland and Altman plot difference between the two researchers’ scores vs, average of the two REALISE scores at T2

The Bland and Altman plot shows the range of agreement between the first and the second researchers' results. In the graph the difference between the scores are plotted against the average of the two measurements. The Bland–Altman plot shows that the differences within mean ± 1.96 SD (− 0.12 + 0.74 and 0.013 + 0.60) are not clinically relevant and the statistical test (*t* test) confirms the mean differences of scores is zero. The degree of agreement between the two researchers’ results is good and the administration of the questionnaire is repeatable since at T2 95% of differences is within two standard deviations (CR in T1 was 1.45, CR in T2 was 1.18) (Figs. 1 and 2).

**Fig. 3:**
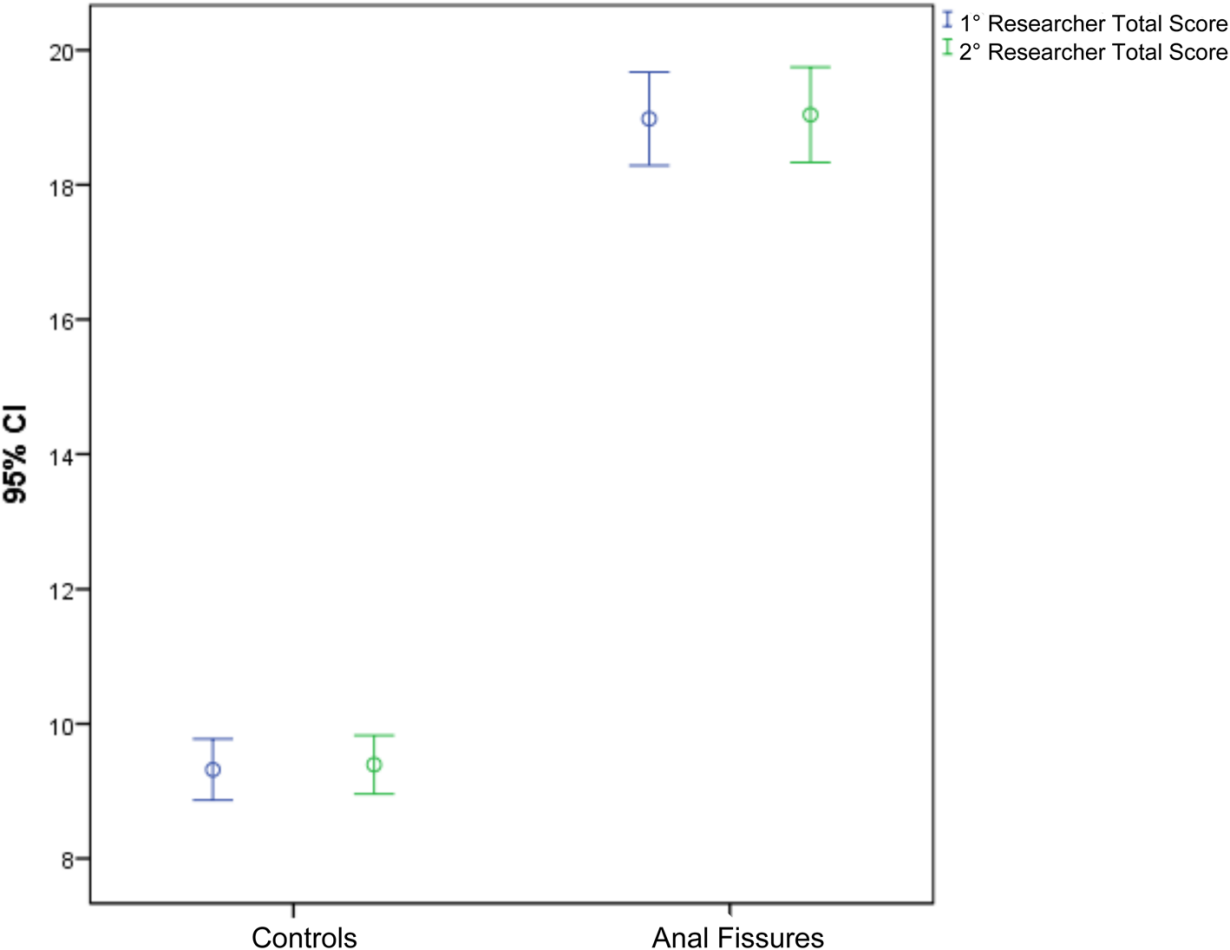
Error bar chart showing the mean and SD of the controls (two researchers) and of the patients with anal fissure (two researchers)

The mean REALISE score after the first administration of the questionnaire was 9.32 (SD ± 2.81) in the control group and 18.98 (SD ± 4.3) in the AF group. After the second questionnaire was administered, the mean value of the REALISE score was 9.39 (SD ± 2.69) in the control group and 19.04 (SD ± 4.38) in the AF group. Therefore, there was a significant difference between the mean REALISE score for patients with AF and controls (*t* = 23.009, *p* < 0.001 and *t* = 22.992, *p* < 0.001) (Fig. 3).

**Fig. 4:**
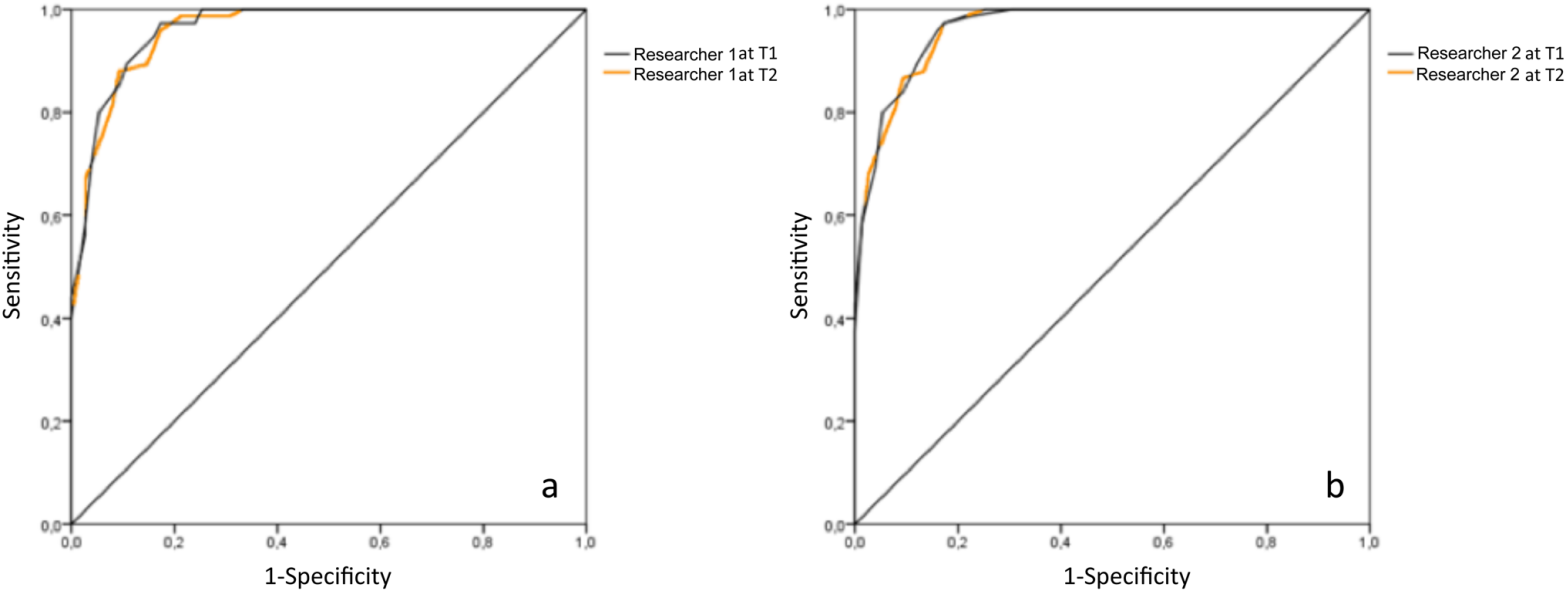
**a**, **b** Receiver operating characteristic curves evaluating the sensitivity and specificity of the scores calculated by the first and second researchers

Both the areas under the ROC curve (AUC) evaluating the sensitivity and specificity of the score obtained by the first researcher at T1 (CI 0.93–099) and at T2 (CI 0.94–0.99) and by the second researcher at T1 (CI 0.94–0.99) and at T2 (CI 0.94–0.99) were 0.96 (Fig. 4a/b) anal fissure.

## Discussion

AF has a negative effect on patients’ quality of life and a significant impact on the public health system. In fact, the main symptom, pain, can impair the social life and ability to work and often leads to refusal to defecate or postponement of defecation because of the fear of intense and prolonged anal pain.

While several symptom-based scoring systems are currently used during the diagnosis and the follow-up of haemorrhoidal disease [13–15], obstructed defecation syndrome [16, 17] and anal fistula [18, 19], there is no validated scoring system for AF. In fact, most of the trials and prospective studies dealing with the efficacy of medical and surgical treatments for AF usually evaluate measures of pain relief, anal bleeding, healing rate and quality of life separately, using non-specific questionnaires such as the EQ-5D health profile [20] and EQ-VAS global assessment of health [21].

A first attempt to evaluate the burden associated with haemorrhoidal disease and AF has been recently proposed by Abramowitz et al. [22]. Nevertheless, the HEMO-FISS questionnaire contains non-specific items and cannot distinguish between haemorrhoidal disease and AF. Furthermore, objective parameters such as the intake of nonsteroidal anti-inflammatory drugs or other painkillers, and bleeding have not been considered.

Based on the most common symptoms and signs in patients with AF and on the concept that an ideal scoring system should be as simple as possible, easy to remember and statistically validated, the REALISE score includes pain assessment evaluated by a VAS for the intensity and by specific questions regarding the duration of pain and the need for painkillers, anal bleeding and changes in quality of life.

The present study has shown that this score has shown an internal and external consistency and has been demonstrated to be repeatable with a high agreement between two different researchers who administered the questionnaires at two different times, and that it is specific for patients with AF. In fact, when the AF group was compared with the control group, the novel scoring system showed a high discriminatory power with a high sensitivity and sensibility in identifying AF patients.

This study has some limitations. It was a single centre study and the questionnaire was administered in a relatively short interval (which is, however, justified by the possible changes of the symptoms severity due to the prescribed therapy). External validation by other research groups would strengthen the interobserver agreement and the applicability of the REALISE score for the assessment of AF symptoms so that this scoring system could be used to select patients for prospective randomised controlled trials on AF to evaluate the outcome of medical and/or surgical treatment.

## Conclusions

This novel statistically validated score for the assessment of AF may have important implications for the assessment and the management of this common and painful condition. It may be a useful tool for grading the severity of the disease and for comparing results of different treatments in future studies on AF.
